# Consumer nutrition environment measurements for nutrient-dense food availability and food sustainability: a scoping review

**DOI:** 10.1186/s13690-023-01231-y

**Published:** 2024-01-15

**Authors:** Katherine Baker, Laura Burd, Roger Figueroa

**Affiliations:** 1 Division of Nutritional Sciences, Cornell University, Ithaca, NY USA; 2 Sol Price School of Public Policy, University of Southern California, Los Angeles, CA USA

**Keywords:** Food environment, Nutrient-dense foods, Sustainable diets, Food sustainability

## Abstract

Consumer nutrition environments are defined as places in which consumers interact with the food they eat; these food choices can impact human and planetary health. Assessment measures for consumer nutrition environments are numerous, and vary widely on what, and how, they assess the food environment. The objective of this scoping review was to synthesize existing evidence on nutrition environment measurements and their capacity to assess nutrient-dense food access and food sustainability capacity. Eligibility criteria were developed to capture relevant peer-reviewed literature about auditing measures designed to assess nutrient-dense foods and food sustainability capacity in the consumer nutrition environment. A search strategy was conducted to collect articles published between January 1, 2002-June 4, 2022, using PubMed, Web of Science, Scopus, PsycINFO and the Cochrane library electronic databases. After screening 2457 manuscripts, 58 met inclusion criteria. Study aims, funding source(s), types of retailers assessed, assessment format and name, constructs measured, food categories measured, assessment of validity and/or reliability, and other relevant data were extracted from each manuscript. Results showed that most measures use checklists, surveys, questionnaires or similar methods to assess availability, quality, and price of select food items as assessment constructs. Most do not assess nutrient-dense food availability, and even fewer assess food sustainability. Development of comprehensive, reliable, and valid consumer nutrition environment measures that assess nutrient-dense food availability and food sustainability is important for research, planning, evaluation and advocacy aimed at improving consumer food environments for human and planetary health.

**Supplementary Information:**

The online version contains supplementary material available at 10.1186/s13690-023-01231-y.

## Background

Suboptimal diets, which contribute to malnutrition and dietary risks, are a leading cause of chronic disease and poor health globally [1–3]. As such, there is a need to prioritize achieving global nutrition security. Nutrition security refers to consistent access to food of sufficient quantity and quality in terms of variety, diversity, nutrient content, and safety to allow people to meet their dietary needs and food preferences for a healthy life [4]. Access to nutrient-dense foods is important for nutrition security, and consuming a diet that reduces the risk of chronic diseases, including Type 2 diabetes, cardiovascular disease, and certain cancers [5]. Nutrient-dense foods are those that provide vitamins, minerals and other health-promoting components with little to no added sugars, saturated fat, and sodium [5]. Unfortunately, access to nutrient-dense foods is threatened by climate change, as climate change and rising levels of carbon dioxide threaten crop yields and nutrient-density [6–8]. Furthermore, suboptimal-diet related health risks are expected to worsen as climate change progresses [6]. To reduce the risk of diet-related chronic diseases, and to protect human and planetary health, a global shift towards sustainable diets is imperative [6, 7, 9–11]. Sustainable diets are those with low environmental impacts which contribute to food and nutrition security, and to a healthy life for present and future generations [12]. Prioritizing this shift is an important shift to ensure access to nutritious, health supporting diets for a growing population within planetary bounds [6, 7, 9–11].

The food environment, or the physical, economic, policy and sociocultural surroundings [13] in which someone makes decisions about the foods they eat, can impact access to and consumption of healthy or nutrient-dense foods [14–16]. The food environment is also a critical place to implement initiatives aimed at supporting sustainable dietary patterns [17]. In the present study, we examine the consumer nutrition sub-environment, where consumers interact with food and its purchasing [14]. The consumer nutrition environment includes assessment of the availability of nutrient-dense food options, price, in-store marketing/promotion, and placement of food items, and availability of nutrition information, which may impact what foods people select to consume and eat [14–16]. Because the consumer nutrition environment is a place where consumers make decisions about which foods they will purchase and consume, these environments offer an opportunity to implement interventions to support sustainable, healthy diets [13, 17]. Consumer nutrition environments hold a high potential for impact, but at present, tend to be less measured than some components of the food environment, as they have a potentially large number of variables to measure [14]. There is a need to optimize food environments, including consumer nutrition environments, to allow for greater nutrient-dense food access and opportunities to consume sustainable dietary patterns [9]. 

To inform research and policy interventions, it is important to establish rigorous, reliable and valid assessment of consumer nutrition environments for assessment and planning, surveillance, research, evaluation and advocacy [18, 19]. However, there is a lack of standard methods for assessing food environments, including consumer nutrition environments [20, 21]. While many food environment measurements exist, very few consider sustainability [21]. Furthermore, there is a lack of validity and reliability data on many measures [22]. This review aimed to summarize literature on existing consumer nutrition environment measurements that measure nutrient-dense foods and food sustainability. The present study aimed to summarize validity and reliability assessments of existing measurements to summarize rigor of existing measures.

## Methods

The Preferred Reporting Items for Systematic Reviews and Meta-Analyses (PRISMA-ScR) extension for scoping reviews was used for planning and presentation of results [23]. The PRISMA-ScR checklist contains 20 essential items plus 2 optional items for good reporting in scoping reviews [23].

### Search strategy

A systematic literature search was conducted using PubMed, Web of Science, Scopus, PsycINFO and the Cochrane library electronic databases. Search strategy terms included “grocery”, “supermarket”, “retailer”, “bodega”, “corner store”, “market”, AND “nutrition environment”, “food environment”, AND “audit”, “assess”, “measure”, AND “sustainable” or “climate”. Specific search strategies used for each database searched can be found in the study protocol as Supplementary file 1. This study included articles published in English between January 1, 2002 to the date of the search, June 4, 2022. The authors found very few research articles about consumer nutrition environment measurement prior to 2007, but selected a search start date of 2002 to ensure any relevant research articles published 20 years prior to the search date were included. Additionally, previous reviews and reference lists of included studies were manually searched, and relevant articles were added accordingly. Covidence software was used to manage abstract and full text screening, and data extraction.

### Inclusion and exclusion criteria

Eligibility criteria were developed to attempt to capture relevant peer-reviewed literature about auditing measures designed to assess consumer food environments in food stores, specifically those that measured the availability of nutrient-dense foods. They were also developed to capture measurements of in-store sustainability practices in select consumer nutrition environments (with an emphasis on supermarkets, grocery stores, or corner stores/bodegas). Studies that included a measurement of assessing nutrient-dense food availability and/or sustainable food practices in consumer nutrition environments, specifically, food retail stores were included, as a primary objective of the study was to summarize tools that assess these constructs. Financial and cultural inclusivity were included as access to affordable and culturally acceptable foods is a key component of a sustainable dietary pattern [12]. Studies that focused on modifications, or establishment of reliability or validity, of existing consumer nutrition environment measures were also included to help provide context of the rigor of existing measurement tools. Exclusion criteria were also applied. Studies focused on measures designed for assessing food retailer types that are not supermarkets, grocery stores, or corner stores/bodegas were excluded (e.g., measurement tools that measured farmers markets, restaurants, etc.), as these tools are functionally different than those that measure grocery stores, supermarkets, and bodegas/corner stores. Measures designed for assessing nutrient dense food availability or food sustainability via analysis of advertisements or using online resources (e.g., Yelp), were also excluded, as the focus of the present study was, primarily, on the in-store experience. Furthermore, measurement tools designed specifically for rural food environments were also excluded, as rural food retail stores may have different assessment needs, and to reduce scope, the present study opted to focus on urban or similar environments. Studies that used geospatial (GIS) approaches to assessing community nutrition environments were excluded from the present study, as its focus is on consumer nutrition environments. Studies published before January 1, 2002 or after June 4, 2022 were not included. Finally, systematic reviews were excluded as most on similar topics have not been published within the past 5 years, limiting their relevance due to the volume of recent publications in this area. Thus, only original research studies were included.

### Screening

The screening process followed the PRISMA extension for scoping reviews [24]. Two members of the research team first independently applied inclusion and exclusion criteria to the title and abstracts to determine eligibility. Researchers applied inclusion and exclusion criteria to full-text articles that were deemed eligible after title and abstract screening. To ensure reliability, the reviewers met to discuss and resolve discrepancies after abstract and title, and full-text screenings. All disagreements between researchers throughout the screening processes were resolved in a group discussion with at least two members of the research team.

### Data extraction

Two researchers independently extracted data from each article related to: the country each study took place in, study aims, funding source, food retailer types measured, assessment tool formats, assessment tool name, whether or not each tool was a modification of an existing tool, constructs assessed by each tool, foods assessed by each tool, total number of items assessed by each tool, measurement of federal food assistance programs, and mentions of validity and reliability assessment. All extraction disagreements between the researchers were resolved in a group discussion. A detailed description of each construct extracted and rationale for extraction, can be found in Supplementary file 2.

### Synthesis of data

Two researchers independently extracted selected data from each manuscript using Covidence. For some categories, such as assessment tool type, and constructs assessed, researchers could select from a list of common options for each data point. If available options were not reflective of data in a manuscript, researchers also had the opportunity to write in answers, verbatim. Researchers had space to fill in other data constructs, including country or countries of study origin, and assessment tool name, verbatim from manuscripts. Other constructs assessed, such as validity and reliability, were answered as a binary (yes/no). Two researchers met to resolve any discrepancies using Covidence software. In the case of any data extraction discrepancy, the research team reviewed each manuscript carefully as a team and determined the most correct or accurate representation of the data to complete the data extraction sheet. Once the final data extraction sheets were agreed upon by the team, the lead researcher reviewed each extraction sheet for completion and accuracy. The agreed upon data was synthesized in a table (see Supplementary file 3).

## Results

**Fig. 1:**
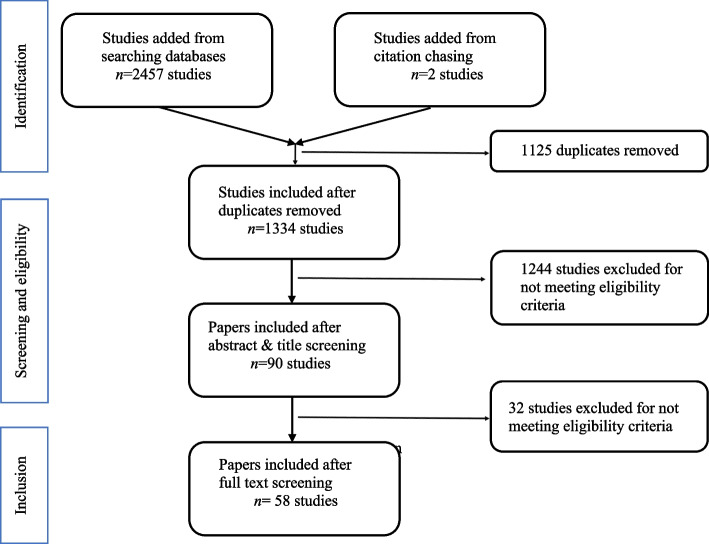
PRISMA flow chart

The search strategy yielded a total of 2459 studies, including 2 studies added from backwards citation chasing. One thousand one hundred twenty-five duplicates identified by Covidence were removed, resulting in a total of 1334 articles for title and abstract screening. During title and abstract screening, researchers determined that 1244 articles did not meet inclusion criteria, and the 90 that did were next screened as full-texts. The most common reasons for exclusion in the final review process included: (1) studies published on existing measures that were not modifications or adaptions of existing measures but rather, utilized a tool already documented in the review without any original contribution, (2) the measurement did not look at consumer nutrition environments, but rather other parts of the food environment, (3) the measurement was made specifically to be used in rural contexts, or (4) the measurement was created to assess food outlets that were not food retailer types listed in the inclusion criteria (e.g., farmer’s market or restaurant assessments). A total of 58 articles were included for data abstraction. Figure 1 provides additional details on the study identification, screening, and inclusion process.

A complete summary chart of data extracted from each manuscript can be found as Supplementary file 3.

### Location

Instruments were developed primarily in the United States (US) (*n* = 37), [25–58], Australia (*n* = 4), [59–62], New Zealand (*n* = 3), [63–65], Canada (*n* = 3), [66–68], Brazil (*n* = 3), [69–71] the United Kingdom (*n* = 2), [72, 73], and Chile (*n* = 2), [74, 75]. Two studies (*n* = 2) were developed to be used in multiple countries [76, 77]. Additional countries examined included China (*n* = 1), [78], India (*n* = 1), [79], South Africa (*n* = 1), [80], and Spain (*n* = 1) [81].

### Assessment method

The most common assessment method was a checklist or similar format (*n* = 36), [25, 26, 28, 32–37, 39–41, 44–49, 51, 52, 56–59, 61, 66–70, 72–74, 78, 79, 81, 82]. Additional assessment methods included the use of a market basket approach which aims to measure foods commonly consumed (*n* = 1), [50], use of an observational form or tool, [29, 71], and assessment of shelf space (*n* = 5) [29, 31, 53, 64, 65, 83]. Other studies used technology, including an electronic store survey, [42], a mobile app (*n* = 1), [60], photo assessments (*n* = 1), [75], wearable cameras, (*n* = 1) [63] and a combination of photo and voice assessment of food environments (*n* = 1) [27]. Some measures used a combination of methods [54, 77].

### Constructs assessed

The majority of measures assessed food availability (*n* = 53), [25, 26, 28–33, 33, 33, 34, 34, 35, 35–37, 47, 48, 50–56, 58–72, 74, 76–83], and food prices (*n* = 36) [25, 26, 29, 32–34, 39, 41, 42, 46–48, 51, 53, 56, 58–60, 66–70, 72–74, 76, 78–83]. Seven studies examined advertisements [34, 55, 69, 70, 76, 83] and 13 examined promotion [39, 43, 53, 59, 60, 62, 63, 65, 70, 71, 73, 76]. Other constructs assessed included variety (*n* = 16) [25, 26, 33, 37, 44, 59, 64–67, 71–74, 81, 83], comparison of healthier vs. less healthy options (*n* = 7), [26, 33, 40, 67, 72, 82] placement (*n* = 9), [39, 43, 55, 59, 60, 62, 63, 69, 73] and accessibility (*n* = 5) [27, 31, 39, 61]. Few studies (*n* = 2) assessed food sustainability [47, 68].

### Foods assessed

Among foods assessed, the most common food categories included fruits (*n* = 45), [25–30, 32–37, 40, 42–47, 49–54, 56–60, 63, 66–71, 73, 75, 77, 78, 80, 82, 83], vegetables (*n* = 44), [25–30, 32–37, 40, 42–47, 49–54, 56–60, 63, 66–69, 71, 73, 75, 77, 78, 80, 82, 83], cow’s milk/dairy, (*n* = 32) [25, 26, 28, 30, 33–35, 37, 39–42, 44, 45, 47, 48, 50–53, 56, 59, 60, 63, 66, 67, 69, 75, 78, 80–82], grains or grain products (such as bread or cereal), (*n* = 24) [25, 33, 35, 39, 42, 44, 46–48, 59, 60, 63, 67, 69, 75, 78, 81, 82], and meat (*n* = 23) [28, 36, 40, 41, 44, 46, 50–53, 56, 59, 60, 63, 66, 68, 69, 75, 78, 80–82]. Other food categories commonly assessed included snack foods (*n* = 17), [29, 40, 45, 49, 53–55, 59, 60, 63, 66, 68, 71, 77, 80], candies (*n* = 5), [29, 39, 43, 54, 71], ultra-processed foods (*n* = 4), [69, 74, 79, 83], sugary beverages or sugar-sweetened drinks, (*n* = 7) [29, 44, 63, 71, 78, 80] and meat alternatives (*n* = 4) [40, 56, 66, 67]. Several studies (*n* = 6) broadly compared healthier or ‘minimally processed’ foods to those that were less healthy or processed [31, 45, 62, 64, 76, 82]. Some studies focused on singular or dual categories of foods, such as junk foods [65] or fruits and vegetables [57, 58]. Fruits and vegetables were among the most commonly assessed food items or food categories. Eighteen studies measured both fresh fruits and fresh vegetables [25, 26, 28, 30, 35–37, 40, 42, 45, 46, 51, 53, 54, 56, 67, 81, 82]. Twenty-two studies assessed fruits, [27, 29, 32–34, 39, 43, 47, 50, 52, 57–60, 66, 68, 71, 73, 77, 78, 80, 83] and vegetables, [27, 29, 32–34, 39, 43, 47, 50, 52, 57–60, 66, 68, 71, 73, 77, 78, 80, 83], without specifying the type (fresh, frozen, canned, etc.). Frozen fruits (*n* = 10) [30, 35, 36, 40, 44, 51, 56, 67, 68, 82], and frozen vegetables (*n* = 15), [25, 30, 35, 36, 40, 44, 51, 56, 67, 68, 82], canned fruits (*n* = 13) [25, 26, 30, 35, 36, 40, 44, 51, 54, 67, 68, 75, 82], and canned vegetables (*n* = 13), [25, 26, 30, 35, 36, 40, 44, 51, 54, 67, 68, 75, 82], were also frequently assessed. One study measured “single” fruits and vegetables [44], one measured dried fruit, [50], and another “all fruits and vegetables” [49]. Four additional studies mentioned assessing “produce” [35, 41, 48, 55].

### Access

Among studies conducted in the US (*n* = 37), [25–30, 32, 34–37, 39–42, 44, 46, 47, 51–53, 56, 59, 62, 65–69, 71–73, 78, 79, 81–83], eight studies collected information on whether or not stores accepted Supplemental Nutrition Accessibility Program (SNAP) [25, 26, 28, 32, 40, 42, 48, 50, 55], and seven collected information on whether or not stores accepted Special Supplemental Nutrition Program for Women, Infants and Children (WIC) [25, 26, 28, 32, 40, 48, 50]. Few studies (*n* = 2) looked at other aspects of accessibility such as physical accessibility [27, 39].

### Measure development and adaptations

Thirty-six studies in the review presented measures that were adaptations of other, existing measures (*n* = 36) [25–30, 32, 34–37, 39–42, 44, 46, 47, 51–53, 56, 59, 62, 65–69, 71–73, 78, 79, 81–83]. For example, some studies would modify or adapt an existing measure to fit a new geographic context or food retailer stores type. The most commonly adapted measure is the Nutrition Environment Measures Survey for stores (NEMS-S) developed by Glanz et al., (2007) (*n* = 20) [25, 26, 28, 34, 35, 37, 40, 46, 52, 53, 56, 66–68, 71, 78, 79, 81–83]. Some measures modified or combined several measures; for example, the FoodNest measure created by Glickman et al., 2021 [34] modified the Nutrition Environment Measurement Score in Corner Stores (NEMS-CS) and the Bridging the Gap Community Obesity Measures Program. Food items assessed ranged from 7 to 196 items in studies that reported items measured.

### Validity and reliability assessment

Among 58 studies included in the final review, 24 mentioned assessing validity of the food environment measures [30–33, 35, 39, 40, 42, 43, 49, 50, 59, 60, 63, 66–71, 73, 78, 81, 82]. Five additional studies mentioned basing their measures off of existing validated studies [28, 41, 52, 68]. Specifically, seven examined construct validity, [33, 43, 59, 65, 69–71] four examined face validity [32, 35, 53, 81] and one examined criterion validity [39]. Thirty-one studies mentioned assessing reliability [25, 26, 28, 30–33, 35, 39–43, 53, 54, 58–60, 62, 65–73, 78, 81, 82]. The most common means of reliability assessment was inter-rater, inter-observer or inter-coder reliability (*n* = 24) [25, 26, 30, 32, 33, 35, 39, 40, 42, 43, 49, 50, 59, 60, 63, 66–68, 70–73, 78, 82].

## Discussion

Given that food represents a key opportunity to protect human and planetary health, and that the consumer nutrition environment represents an important opportunity to improve access to nutrient-dense and sustainable foods [17], robust and comprehensive measures of these environments are important [84, 85]. This review aimed to summarize literature on existing consumer nutrition environments measures, including access to nutrient-dense foods, food sustainability practices, and reliability and validity. Regarding study aims, many studies included in this review aimed to develop or validate existing tools, to assess, describe, document, or compare food environments. Other aims included measurement of specific foods (e.g., fruits and vegetables), or to assess healthiness of food environments. Many measures exist, including many checklist or similar formats (i.e., questionnaire), shelf space assessments, market basket approaches, and some technology-enhanced methods (i.e., mobile apps). Constructs frequently measured include availability, price, quality, variety, placement, accessibility, and comparison of healthy vs. less healthy food choice options. Only two studies had any assessment of environmental sustainability. Regarding foods assessed, almost all studies included in this review measured fruits and vegetables. Other foods assessed included cow’s milk/dairy, grains or grain products, meats, snack foods, sugar-sweetened drinks, and candies or ultra-processed foods. Thirty-six measures were adaptations or modifications of other measures. The most commonly assessed food retailer types included convenience stores (*n* = 31), supermarkets (*n* = 29), and grocery stores (*n* = 28), with other food retailer types including corner stores (*n* = 5) and dollar stores (*n* = 4). Of the 58 studies included in the review, 24 assessed validity, and 31 assessed reliability.

Many studies measured “healthy” foods or food items. The definition of healthy varied by study, and some did not specify or clarify what criteria was used to define foods as healthy. This makes comparison of findings across various consumer nutrition environment assessments challenging. The authors hence suggest future consumer nutrition environment measures clearly define criteria for categorizing foods as healthy or nutrient dense. For example, the definition of nutrient-dense could follow the definition in the most recent Dietary Guidelines for Americans (DGAs) [5], or the Food and Drug Administration’s definition of “healthy” for food labeling could be used [86]. While these two definitions differ slightly, they exemplify potential means of systematically categorizing foods according to objective metrics, such as sodium and added sugars content.

While many studies measured availability of healthy foods, only two studies included any assessment of environmental sustainability. Specifically, a study by Lupolt and colleagues examined availability of sustainable food choices, food waste, packaging reduction, availability of organic foods, milk produced without hormones or antibiotics, grass-fed milk and plant-based milk [47]. Mollaei and colleagues developed a measure assessing availability of foods available to achieve a low-carbon dietary pattern based on what is available to Ontario residents [68]. The latter is more in line with major efforts to shift towards sustainable diet patterns that emphasize plant-based diets and away from high meat consumption, particularly ruminant animal crops like beef and lamb, for overall food sustainability [9]. While these measures acknowledge the importance of examining food sustainability as part of food environment assessment, research gaps remain on how much certain aspects of various production measures, such as organic agriculture, matter in terms of overall sustainability of a food product [87], which may limit current attempts to quantify food sustainability capacity in consumer nutrition environment settings.

In addition to increasing standardization for measuring nutrient-dense foods and sustainable foods, there is also opportunity to determine appropriate scope and method for consumer nutrition environment measures. The large range of items or varieties of food types assessed (7 to 196) indicates marked differences in the scope of existing measures. Given that a seven-item measure was found to have comparable validity with the original NEMS-S [52], it may be worth exploring what number of measurement items is considered adequate to measure consumer nutrition environments, as, at present, the optimal scope of assessment that meets research needs while maintaining logistical feasibility remains unclear. Regarding methodology, results of the current review show that many studies measured entire food categories by a single or a few specific foods. For example, NEMS-S and other studies assess availability of fruits and vegetables based on whether a store has a checklist of items, such as apples and carrots [33]. While measuring selected foods may be useful in some food retail stores, surveying specific foods as proxies for larger food categories has the potential to miss other foods that may be available to build nutrient-dense and sustainable food patterns, especially across geographic and cultural contexts. There are several measures that assess foods relevant to certain geographical or cultural contexts, including specific cities, states, or cultural food patterns [26, 30, 35, 36, 67, 81]. Future measures may build upon these tools, and aim to broadly assess overall nutrient-dense food availability and food sustainability capacity across cultural contexts. Lastly, regarding rigor, the present study found 54.44% of the 58 studies were assessed for reliability and 41.38% for validity. A 2017 systematic review focused on food environment assessment reported that 25.9% of tools measuring the food environment assessed reliability, and 28.2% reported validity [84]. Establishing validity and reliability is important for ensuring data are replicable and results are accurate [88]. There is thus an overall need for improved reliability and validity assessment of food environment tools, including consumer nutrition environment tools [89] to improve measurement capacity and rigor [18, 19, 90].

Overall, the results of the current review suggest a wide range of consumer nutrition environment measures. They also highlight opportunities to improve systematic measures of both nutrient-dense foods and sustainability capacity, and the importance of considering cultural context and inclusivity. These findings align with those of a 2012 systematic review of consumer nutrition audit tools [84], suggesting continued room for improvement the consumer nutrition assessment space. Measures in this review were also heterogeneous, making it difficult to draw conclusions across studies. This limitation is not unique to consumer nutrition environment measures, but exists across food environment assessment as whole [19, 84]. Enhanced reliability and validity may help to increase the rigor of existing and future measurements. Furthermore, while the field of food environment measurement has collected copious amounts of data, there is still no consensus on best ways to manage or utilize the data (82). Future efforts may establish best practice for managing, analyzing, and interpreting consumer nutrition environment data.

This study has several limitations. First, as a scoping review, critical appraisal of evidence quality is not a requirement and was not conducted [91]. This is a limit as it does not identify gaps in literature that may exist due to low quality [91]. Additionally, as a scoping review, it is not exhaustive or comprehensive, but rather assesses an area of inquiry, in our case, consumer nutrition environment assessment of nutrient-dense food availability and food sustainability capacity [91]. This research is intended to map key concepts to inform future systematic reviews and/or research [91]. Thus, this review alone is not a complete and representative example of all aspects of consumer nutrition assessment. It excluded literature about tools that measure rural consumer nutrition environments, or other aspects of the consumer nutrition environment, including those tailored to other food retailer types, such as farmers markets or restaurants, which may play important roles in the lives and diets of consumers. Lastly, studies outside of the electronic databases used in this review may have been missed. Despite these limitations, this review offers a systematic search strategy completed in five databases that cover a range of health and public health-related subject areas, including those related to nutrition and sustainability. Furthermore, the application of the PRISMA-ScR guidelines to the planning and dissemination of the review add rigor to the scoping review methodology adopted [24]. Finally, this study contributes to existing consumer nutrition environment literature by adding a sustainability component, which is critical to support efforts on nutrition and food security, as well as planetary health going forward.

## Conclusions

Many consumer nutrition environment measures exist, with range in scope, and constructs assessed. Most commonly, consumer nutrition environment measures assessed availability, price, quality, variety, placement, accessibility, and comparison of healthy vs. less healthy food choice options, and only two measures had any mention of environmental sustainability. Furthermore, many studies lack reliability and validity. There is opportunity to improve consumer nutrition environment assessment with validated, reliable measures that utilize recent data on nutrient-dense foods and food sustainability capacity. Such measures will help public health researchers, practitioners and policy makers for research, planning, evaluation and advocacy, targeting improved nutrient-dense food availability and food sustainability capacity in consumer food environments.

### Supplementary Information

**Additional file 1.**

**Additional file 2.**

**Additional file 3.**

## Data Availability

Not applicable.

## Abbreviations

PRIMSA: Preferred Reporting Items for Systematic Reviews
PRISMA-ScR: Preferred Reporting Items for Systematic Reviews and Meta-Analyses
FFAP: Federal food assistance program
NEMS-S: Nutrition Environment Measures Survey Supermarkets
SNAP: Supplemental Nutrition Assistance Program
WIC: Women, Infants and Children
