# Supplementary material for: Consumer nutrition environment measurements for nutrient-dense food availability and food sustainability: a scoping review
Source: Arch Public Health. 2024 Jan 15;82:7. doi: 10.1186/s13690-023-01231-y (PMC10789067; doi:10.1186/s13690-023-01231-y)
Supplement: Supplementary file 1 — Additional file 1. [file 13690_2023_1231_MOESM1_ESM.docx]

**Review Question:** What is known from existing literature about food retailer auditing tools for assessing nutrient-dense food availability and sustainability practices?

Search strings used.

Searches completed on June 4, 2022.

| **Database searched** | **Search string** |
| --- | --- |
| SCOPUS | TITLE-ABS-KEY ((grocer* OR supermarket* OR retailer* OR bodega* OR "corner store*" OR market*) AND ("nutrition environment" OR "food environment") AND (audit* OR assess* OR sustain* OR climate* OR *other synonyms*)) AND |
| Web of Science | TS=((grocer* OR supermarket* OR retailer* OR bodega* OR "corner store*" OR market*) AND ("nutrition environment" OR "food environment") AND (audit* OR assess* OR sustain* OR climate* OR *other synonyms*)) |
| PsychINFO | TI=((grocer* OR supermarket* OR retailer* OR bodega* OR "corner store*" OR market*) AND ("nutrition environment" OR "food environment") AND (audit* OR assess* OR sustain* OR climate*  OR  AB = ((grocer* OR supermarket* OR retailer* OR bodega* OR "corner store*" OR market*) AND ("nutrition environment" OR "food environment") AND (audit* OR assess* OR sustain* OR climate* OR *other synonyms*))  OR  KW = ((grocer* OR supermarket* OR retailer* OR bodega* OR "corner store*" OR market*) AND ("nutrition environment" OR "food environment") AND (audit* OR assess* OR sustain* OR climate* OR *other synonyms*)) |
| Cochrane library | ((grocer* OR supermarket* OR retailer* OR bodega* OR "corner store*" OR market*) AND ("nutrition environment" OR "food environment") AND (audit* OR assess* OR sustain* OR climate* OR *other synonyms*)) |
| PubMed | ((supermarket[MeSH] OR grocer*[tiab] OR supermarket*[tiab] OR retailer*[tiab] OR bodega*[tiab] OR "corner store*"[tiab] OR market*[tiab]) AND (Access to Healthy Foods[MeSH] OR "nutrition environment"[tiab] OR "food environment"[tiab]) AND (audit*[tiab] OR assess*[tiab] OR climate* OR sutain* *other synonyms*) |

**Article title:** Food retail environment audits for measuring nutrient-dense food availability and food sustainability: A scoping review

**Authors names and affiliations:** Katherine Baker^1^, MS, MPH, Laura Burd^1^, Roger Figueroa^1^, PhD, MPH, MSc

^1^ Division of Nutritional Sciences, Cornell University, Ithaca, New York, United States

**Correspondence**: Katherine Baker, [kb739@cornell.edu,](mailto:kb739@cornell.edu) ORCID: 001-6739-077

**Journal name:** Achieves of Public Health
