# Supplementary material for: Consumer nutrition environment measurements for nutrient-dense food availability and food sustainability: a scoping review
Source: Arch Public Health. 2024 Jan 15;82:7. doi: 10.1186/s13690-023-01231-y (PMC10789067; doi:10.1186/s13690-023-01231-y)
Supplement: Supplementary file 2 — Additional file 2. [file 13690_2023_1231_MOESM2_ESM.docx]

| **Data construct extracted** | **Definition and means of extraction** | **Extraction rationale** |
| --- | --- | --- |
| Country | Reviewers wrote in the country or countries verbatim. | Understanding what geographical context a measurement was designed for or tested in can help inform where it is best to use. |
| Study aims | Reviewers listed study aims or objectives listed by authors verbatim on the data extraction sheet. Study aims or objectives state what authors of each study describe the main goals or purpose of the research study. | Study aims help contextualize the purpose of the measurement tool and its intended use. |
| Funding source | Data about the source of funding for each research study was extracted verbatim, if it was listed in the manuscript. | Funding sources can influence or bias research (19) awareness of a study’s funding source can help readers understand these potential influences. |
| Food retailer types measured | Reviewers completed a checklist identifying which types of retailers the tool was intended for, including bodega/corner store/convenience stores, supermarkets, and also had an “other” option, where the reviewer could write in the food retailer type, verbatim. | Different consumer nutrition environment measurement tools may be intended for, or work best for, certain retailer types. Understanding the type of stores each were intended for can help readers |
| Assessment tool format | Constructs assessed by the nutrition environment measurement tool were extracted; for example: availability, advertisements, nutritional information, price, placement, promotion, quality, variety, sustainability, etc.; reviewers could also write in other constructs verbatim. | Understanding the formats of different measurements used can help inform development of future measurement tools. |
| Assessment tool name | The name of each tool, if listed by the authors, was extracted verbatim. | Tool identification/recognition. |
| Modification of existing tool | Data was extracted in a binary (yes/no) to document if the tool in present article was a modification or adaption of an existing tool. | Many consumer nutrition assessment tools are iterations of other assessment tools; quantifying how many helps gauge the scope of how many novel vs. iterations of other tools exist in literature. |
| Constructs assessed by tool | Constructs assessed by the nutrition environment measurement tool were extracted; for example: availability, advertisements, nutritional information, price, placement, promotion, quality, variety, sustainability, etc. Reviewers listed constructs assessed by authors verbatim to avoid interpolation of what was extracted. | Understanding constructs commonly assessed can inform future tools. Furthermore, understanding under measured aspects of the consumer nutrition environment can inform future tool development. |
| Food categories assessed by tool | Food types or categories assessed by the tool were extracted. For example, common food categories extracted included fruits, vegetables, milk, cheese, bread, grains, etc. Reviewers listed constructs assessed by authors verbatim to avoid interpolation of what was extracted. | Quantifying most and least commonly measured food categories and groups can help identify important foods to measure and gaps in literature that could be assessed in other studies. |
| Total number of items assessed by tool | The number of items measured by the food environment assessment tool was extracted, if listed by the author. If the number was not listed by the author and a complete version of the auditing tool was available in the manuscript, as a supplementary file, or by contacting the author, the number of items assessed was counted. If the number was not listed in the manuscript or a copy of the tool was not available, this item was marked as “N/A”. | Understanding the range of items measured and being able to compare their validity and reliability (see below) can help inform the scope future measurement tools. |
| Federal food assistance program (FFAP) acceptance | Reviewers completed a checklist for federal food assistance programs assessed by measurement tool, including the Supplemental Nutrition Assistance Program (SNAP) and the Special Supplemental Nutrition Program for Women, Infants, and Children. This was not applicable to study tools designed for use in other countries. | FFAP such as SNAP can enhance food security and improve health outcomes(20). Understanding how many tools assess this component of financial accessibility can inform how robustly this is being accounted for in consumer nutrition environment studies. |
| Validity | Validity refers to how well the study findings represent the truthfulness of the findings (21). Validity was extracted as a binary (yes/no) based on whether authors of each study mentioned establishment of validity. If authors mentioned a kind of validity measured (construct, etc.) reviewers wrote in this information, verbatim, as well. | Understanding the validity of a measurement tool can help understand its strengths and limitations. |
| Reliability | Reliability refers to the stability of findings (or how reproducible results are with the same conditions. Reliability was extracted as a binary (yes/no) based on whether authors of each study mentioned establishment of reliability. If authors mentioned a kind of reliability measured (inter-rater, intra-rater, etc.), reviewers wrote in this information, verbatim, as well. | Understanding the validity of a measurement tool can help understand its strengths and limitations. |
